# The Development and Piloting of a Mobile Data Collection Protocol to Assess Compliance With a National Tobacco Advertising, Promotion, and Product Display Ban at Retail Venues in the Russian Federation

**DOI:** 10.2196/resprot.5302

**Published:** 2016-08-31

**Authors:** Ashley S Grant, Ryan D Kennedy, Mark H Spires, Joanna E Cohen

**Affiliations:** ^1^ Institute for Global Tobacco Control Department of Health, Behavior & Society Johns Hopkins Bloomberg School of Public Health Baltimore, MA United States

**Keywords:** tobacco, tobacco marketing, retail environments, compliance assessment, policy implementation, point-of-sale, Russia, mobile data collection, mobile devices

## Abstract

**Background:**

Tobacco control policies that lead to a significant reduction in tobacco industry marketing can improve public health by reducing consumption of tobacco and preventing initiation of tobacco use. Laws that ban or restrict advertising and promotion in point-of-sale (POS) environments, in the moment when consumers decide whether or not to purchase a tobacco product, must be correctly implemented to achieve the desired public health benefits. POS policy compliance assessments can support implementation; however, there are challenges to conducting evaluations that are rigorous, cost-effective, and timely. Data collection must be discreet, accurate, and systematic, and ideally collected both before and after policies take effect. The use of mobile phones and other mobile technology provide opportunities to efficiently collect data and support effective tobacco control policies. The Russian Federation (Russia) passed a comprehensive national tobacco control law that included a ban on most forms of tobacco advertising and promotion, effective November 15, 2013. The legislation further prohibited the display of tobacco products at retail trade sites and eliminated kiosks as a legal trade site, effective June 1, 2014.

**Objective:**

The objective of the study was to develop and test a mobile data collection protocol including: (1) retailer sampling, (2) adaptation of survey instruments for mobile phones, and (3) data management protocols.

**Methods:**

Two waves of observations were conducted; wave 1 took place during April-May 2014, after the advertising and promotion bans were effective, and again in August-September 2014, after the product display ban and elimination of tobacco sales in kiosks came into effect. Sampling took place in 5 Russian cities: Moscow, St. Petersburg, Novosibirsk, Yekaterinburg, and Kazan. Lack of access to a comprehensive list of licensed tobacco retailers necessitated a sampling approach that included the development of a walking protocol to identify tobacco retailers to observe. Observation instruments were optimized for use on mobile devices and included the collection of images/photos and the geographic location of retailers. Data were uploaded in real-time to a remote (“cloud-based”) server accessible via Internet and verified with the use of a data management protocol that included submission of daily field notes from the research team for review by project managers.

**Results:**

The walking protocol was a practical means of identifying 780 relevant retail venues in Russia, in the absence of reliable sampling resources. Mobile phones were convenient tools for completing observation checklists discretely and accurately. Daily field notes and meticulous oversight of collected data were critical to ensuring data quality.

**Conclusions:**

Mobile technology can support timely and accurate data collection and also help monitor data quality through the use of real-time uploads. These protocols can be adapted to assess compliance with other types of public health policies.

## Introduction

### Tobacco Control Policy

Tobacco use is the leading preventable cause of premature death and disease in the world, killing approximately 6 million people annually and costing more than half a trillion US dollars in health care costs [1]. The health burden of tobacco use is heavier in certain countries and regions of the world due to higher prevalence of use. One of the highest burdened countries in the world is the Russian Federation (Russia). The World Health Organization (WHO) estimates that over 60% of men and 22% of women in Russia smoke cigarettes (approximately 43.9 million adults) [2]. The tobacco industry strives to increase tobacco consumption among current users, attract new consumers, and encourage former customers to resume tobacco use through the use of tobacco advertising, promotion, and sponsorship (TAPS) strategies [3]. The tobacco industry spends tens of billions of US dollars worldwide each year to develop TAPS strategies, produce marketing media, and advertise their brands [1,4]. Exposure to tobacco advertising and the availability of promotions in the retail environment are known to increase the likelihood of smoking uptake among youth [5]. Article 13 of WHO’s Framework Convention on Tobacco Control recommends the adoption and implementation of comprehensive laws that ban all forms of TAPS [6]. Complete bans on TAPS activities that prohibit the display of products (in addition to signage and other promotions) at the point-of-sale (POS) are essential policies that reduce people’s exposure to tobacco marketing [7]. Although TAPS bans that eliminate POS marketing are a crucial element of comprehensive tobacco control strategies, even jurisdictions with strong community support face significant barriers to successful implementation. These policies can be challenged by tobacco companies on the basis of laws that govern freedom of expression and free enterprise [8].

In Russia, the federal law N 15-FZ “On Protecting the Health of Citizens from the Effects of Second Hand Tobacco Smoke and the Consequences of Tobacco Consumption” [9] (2013), addresses such tobacco control measures as smoke-free places, TAPS bans, packaging and labeling, price, tax, and sale. On June 1, 2013, the sale of tobacco products was prohibited in the premises and within 100 meters of locations that provide educational services. The law later banned all forms of advertising, promotion, and sponsorship effective November 15, 2013 including signage, distribution of free products, use of price discounts, brand stretching, use or imitation of tobacco products, organization and performance of events (such as concerts), and the use of tobacco trade names in charitable activities. The law prohibited the display of tobacco products at “retail trade sites” [9], effective June 1, 2014. Retail venues are permitted to provide customers with an alphabetized list of products and prices in plain black font on a white background, with no graphics or images. Further regulations took effect on June 1, 2014, prohibiting the sale of tobacco at kiosks (street stalls) [9].

### Monitoring Tobacco Advertising

Rigorous and well-timed tobacco control policy compliance assessments can determine whether the new laws are being followed and can be used to expose loopholes in weak regulations, or to strengthen enforcement [10]. Ideally, assessments of new policies, such as POS advertising, promotion, and display restrictions should be conducted both before and after the law takes effect, within a reasonable implementation period [11]. Sampling should include relevant tobacco retailers and observers should revisit the same retail location in each wave of data collection. A representative sample of tobacco retailers provides the most reliable evidence to inform policymakers and enforcement authorities; however, many jurisdictions do not have complete lists of retailers or such information is not available to nongovernmental organizations (NGOs) or academics interested in policy evaluation. Furthermore, assessments collecting repeat measures from the same locations can be challenging when addresses are poorly labeled or, in the case of Russia, when retail venues are clustered kiosks which may not have an address at all.

City neighborhoods may also differ across relevant dimensions, such as retail offerings and enforcement activities. Retailers tend to cluster and venues will stratify, for example, some parts of a city will have a higher concentration of expensive or high-end retailers, while other neighborhoods will have a higher proportion of low-end or less expensive retail offerings, which must be addressed when selecting a sample of venues. Previous studies have developed retail venue sampling protocols that use a walking pattern to traverse a city while collecting littered tobacco packs for later coding and analysis [12]. Similar work relied on census data to identify and select the neighborhoods within a city from which to collect littered packs [13].

Policy compliance measures should be collected by staff that are knowledgeable about the law in order to ensure the accuracy of observations. Data collectors also need to be suitably discreet to avoid retailer interference, which can be difficult when entering a POS with a clipboard, camera, and global positioning system (GPS) device. Further, the oversight of data collection and data management/validation can be challenging when the sample is geographically dispersed in multiple time zones with thousands of potential retail locations. As with any research study, procedures for communicating with the study team and managing collected data should be in place to verify that the protocol is followed faithfully and that data are collected accurately.

The increasing ubiquity of mobile phones and access to wireless networks makes these devices a realistic option for data collection. Mobile phones and mobile software applications (apps) allow users to collect and record observational data, images, and metadata (such as timestamp, GPS-based location coordinates, device identification number, etc) in the field, using specific or customized survey instruments. Photos are a useful source of data that can be utilized for quality control and validation purposes, for the practical purpose of revisiting certain locations, for research involving secondary analysis of the photos, and as visual representations of the data to assist with dissemination of findings. Additionally, devices that are connected to a network service through cellular data or wireless local area network (WiFi) can upload data immediately, allowing for real-time data validation and project management. Metadata can be cross-referenced to ensure that all technology is functioning properly (device, software, and network) and that data collectors are following the study protocol and schedule.

Mobile technology has been used extensively in public health research and practice, and has proven effective in the collection of data in various settings and locales [14-18]. Mobile phones specifically have been used for data collection whether via SMS/texting or via a mobile Internet connection [19,20]. Interest in using mobile devices in the field of tobacco control is growing, with more work focusing on smoke-free environments, retail environments, health communication, detection of illicit trade products, and support of alternative livelihood options for tobacco farmers and workers [21-23]. One study developed protocols to use mobile phones to collect photos and geospatial data to examine POS marketing characteristics and their effect on craving to smoke; this work relied on sophisticated software platforms and required significant time and human resources to process and code data [24].

Recent work has aimed to mitigate the bottleneck created by the mobile collection of observation data, geospatial data, and photographs and the time required to properly link, code, and annotate these data with the use of crowdsourcing methodology [25]. Crowdsourcing is a convenient option when potential workers are readily available and can be easily dispatched to a clearly defined sampling area to complete a simple task, but may not be conducive to rapid policy assessment and reporting. Procedures or observations that are more complex require significant oversight to ensure adherence to the study protocol, to validate observations, and to troubleshoot technical or logistical issues that arise in the field, which can be difficult to manage among a group of crowdsourced data collectors. Such protocols that also necessitate discretion on the part of the study team in order to avoid interference from the industry are better suited for a group of trained data collectors. Studies show that mobile devices are useful tools for conducting public health research, although the use of such technology entails an additional layer of complexities and limitations that researchers must account for. There remains a need for additional guidance on adapting these tools and literature on the utility of mobile technology for broader public health based research, such as policy implementation.

This manuscript outlines a research protocol utilizing mobile technology to collect observational data before and after the implementation of sales restrictions and a product display ban at tobacco retail venues in Russia. Although the protocol was developed specifically for Russia, the methods are adaptable to other jurisdictions. The protocol includes three components: (1) a rigorous sampling protocol, (2) data collection instruments including capture of images/photos and geographic coordinates, and (3) data management protocols.

## Methods

### Groups Involved in the Study

This work was conducted by the US Institute for Global Tobacco-based Control (IGTC) at Johns Hopkins Bloomberg School of Public Health. IGTC partnered with the Campaign for Tobacco Free Kids (CTFK), an international public health NGO, and a team of Russian tobacco control experts based in Moscow, Russia. Local experts provided guidance and context about the sampling framework, instrument development, and data collection logistics. The in-country team conducted the fieldwork and submitted daily reports for review by the project management team in Baltimore, Maryland.

### Sampling Approach

#### Sampling Protocol Objectives

The sampling protocol first identified which Russian cities would be included in the study. The study team wanted to conduct sampling in socioeconomically diverse areas, so it was necessary to identify zones of the city that met different criteria. The team also needed to identify where observations would occur within those zones (ie, sampling areas) to evaluate implementation and compliance with the law. The sampling strategy considered which tobacco-retailer types (kinds of stores) would be sampled and how to identify those retailers in a manner that was both systematic and sufficiently random, while allowing for timely collection of data with a preference for walking between retailers. Finally, the sampling protocol needed to support a multi-wave design to ensure revisiting retail venue locations would be possible.

The goals of the sampling protocol, the criteria or process used to achieve the sampling goals, and the decisions made are outlined in Table 1. Further details about each step are discussed later.

**Table 1:** Sampling decisions.

Goals		Criteria or process used	Decision
**Identify cities where sampling will take place.**		Include cities that have a large population; include cities that are geographically dispersed throughout the country.	The following five cities were identified: Moscow, St. Petersburg, Novosibirsk, Yekaterinburg, and Kazan.
**Identify diverse socioeconomic zones in each city.**			
	Identify and map zones in city based on socioeconomic status.	Include areas of different socioeconomic status; without available census data on resident education and income, a proxy value of property value is used.	Study mapped 3 types PVZ^a^. PVZ^a^ was classified as being: •High property value (>1.25x average property value) •Average property value (>0.85-1.25 x the average property value), or •Low property value (<0.85x the average property value).
**Identify sampling areas.**			
	In each property-value-zone, identify areas of the city with significant retail activity (retail centers). Create a catchment area around the retail center, this area is known as the sampling area.	Identify three different types of retail centers in each PVZ^a^: 1. Shopping mall, 2. Major intersection, and 3. Transit station.	From each retail center, a 3 km radius was drawn, creating 3 different sampling areas in each of the 3 property-value-zones. [9 sampling areas in each city]
**Identify retail venues to be sampled in each sampling area.**			
	Identify which types of tobacco retail venues (POS) to be included.	Chain supermarkets [both up market (luxury) and mid-low market]; independent markets/convenience stores; Kiosks (street stalls).	Sampling goals included an equal number of: 1. Chain supermarkets, 2. Independent markets/convenience stores, and 3. Kiosks. Chain supermarkets included equal number of luxury/high market stores, and mid-low market stores when possible. Independent markets include both convenience stores and gas stations. [6 chain supermarkets, 6 independent markets, and 6 kiosks per sampling area-18 total retail venues per sampling area]
	Identify locations of specific retailers within the sampling areas.	Use available databases that list and include the location of chain supermarkets (both high end, and mid and low end) in each study city. Map locations of chain supermarkets to identify which stores are in the physical sampling areas. If there are more than 9 chain supermarkets in the sampling area, number stores and use a number generator to randomly identify stores to be included in the sample.	In each sampling area, 6 chain supermarkets were identified and mapped in each of the cities' 9 sampling areas. An additional 3 “back-up” chain super markets were also included in the sampling areas. [54 chain supermarkets per city, and 27 back-up chain supermarkets]
	Identify other retailers nearby supermarkets.	There are no available lists of licensed retailers, so a walking protocol is used to identify near-by independent markets (convenience stores and gas stations) and kiosks.	After data are collected at a chain supermarket, data collectors will exit the supermarket and, using the walking protocol, identify a nearby independent market and kiosk. [54 independent markets and 54 kiosks per city]

^a^PVZ: property-value-zones

#### Cities Included in the Sample

**Figure 1:**
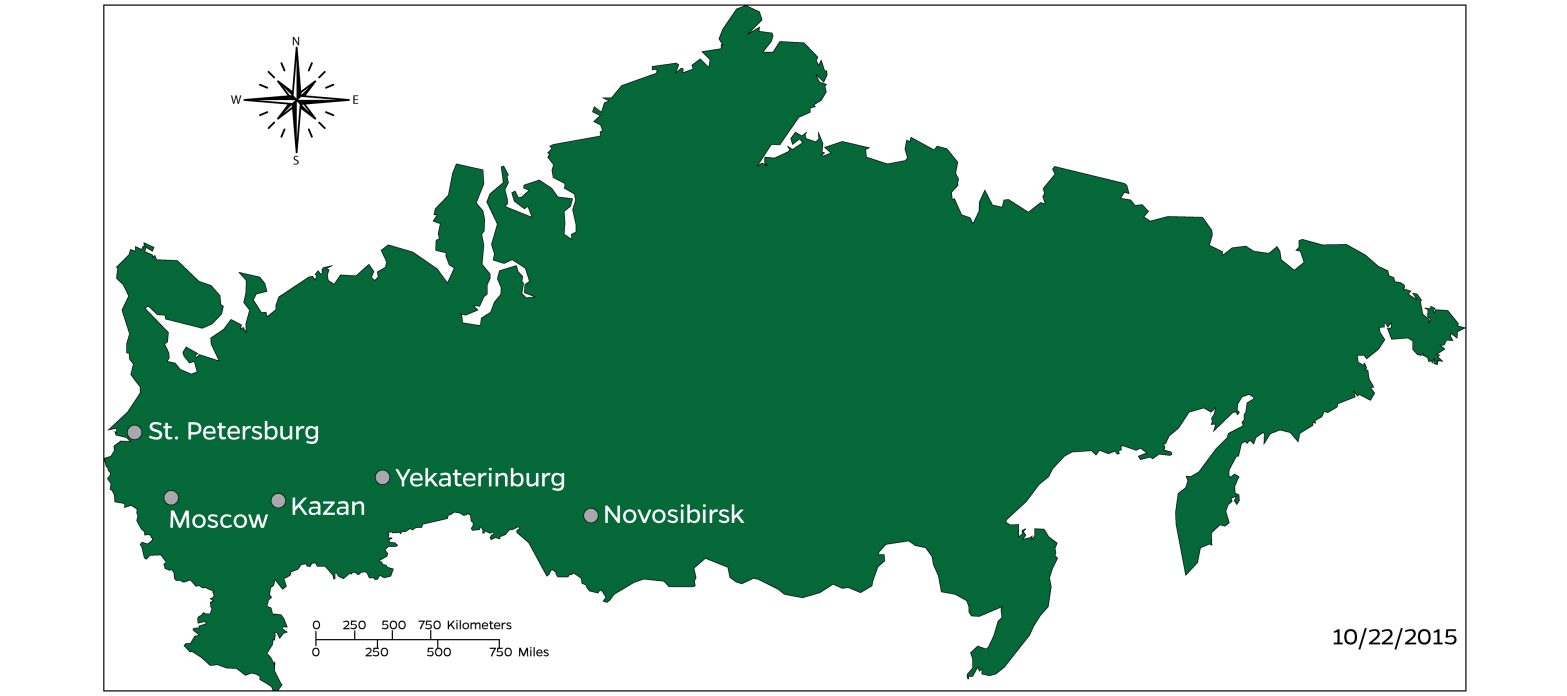
Map of five cities included in the sample.

The cities of Moscow, St. Petersburg, Novosibirsk, Yekaterinburg, and Kazan were selected for data collection based on their relative population (Table 2), and geographical dispersion (Figure 1 shows this). Each of the five cities are located in separate and distinct federal subjects (or municipalities) and are among the top ten most populous cities in Russia.

**Table 2:** Cities included in the sample and their relative rank by population within Russia [26].

City	2012 population size (millions)	Rank (within Russia)
Moscow	11.92	1
St. Petersburg	4.99	2
Novosibirsk	1.51	3
Yekaterinburg	1.39	4
Kazan	1.17	8

#### Property-Value-Zones Identified

**Figure 2:**
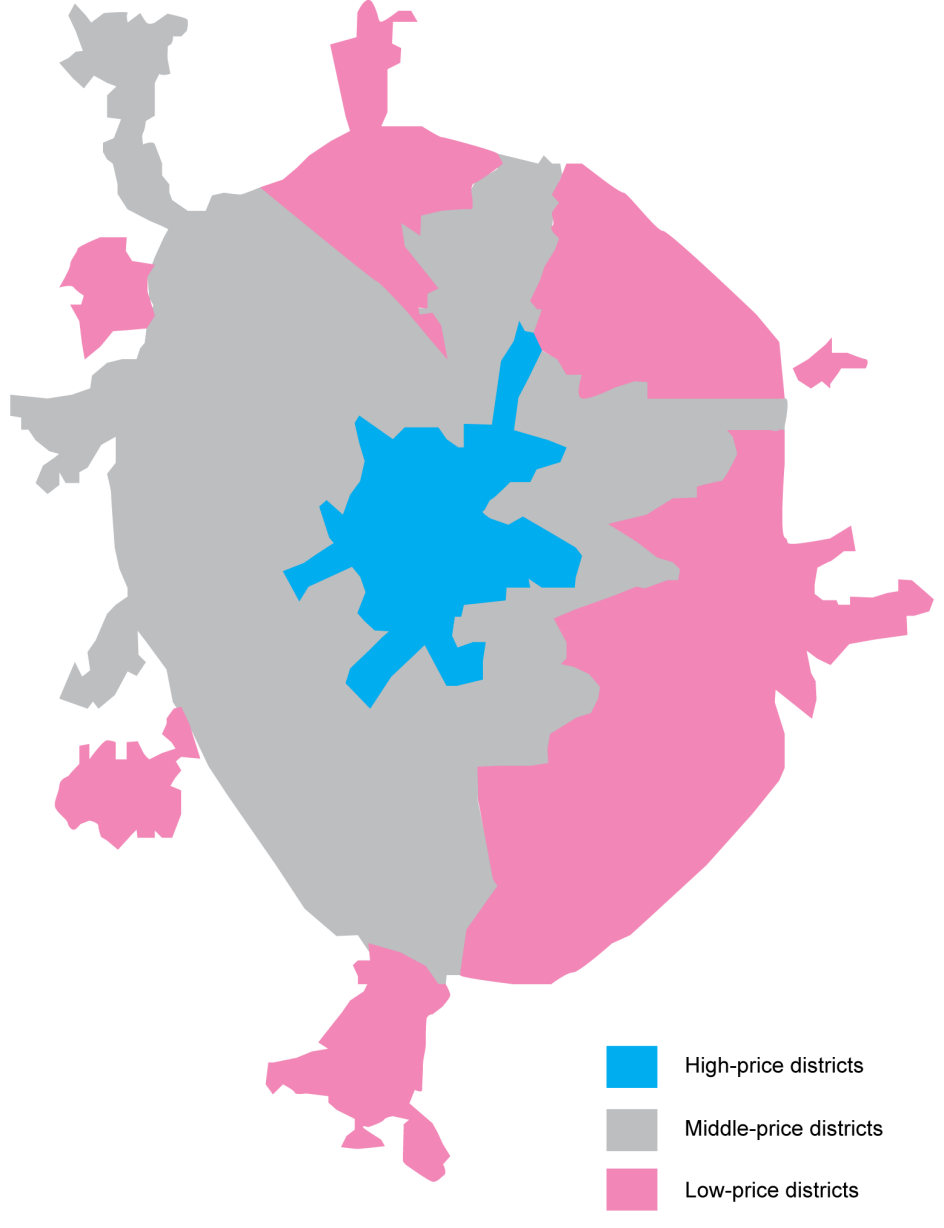
Moscow property-value-zones.

Ideally, census data would be used to identify areas or zones within a city representing a range of socioeconomic statuses (SES). This approach ensures that the evaluation is not conducted in homogenous areas, thereby possibly missing compliance or enforcement trends. Local-level SES data were not available to the research team so a proxy measure, relative real-estate value (rubles per square meter), was used to classify property-value-zones (PVZ). Local experts gathered information about residential property values from reputable sources [27-32]. For each of the 5 cities, maps were created with polygons identifying PVZ’s as “high” (more than 125% of the average real estate price), “average” (85%-125% of the average price), or “low” (less than 85% of the average price). These zones were mapped using Adobe Illustrator (Figure 2 shows this).

#### Identification of Retail Centers and Surrounding Sampling Areas

In each high, medium, and low PVZ, 3 different retail centers were identified including a large shopping mall, a major intersection, and a transit station (subway, bus, or train) for a total of 9 unique retail centers per city. Local experts suggested these types of retail centers based on knowledge of venue dispersion and walkability. Researchers applying this protocol in other settings should consider the retail venue density and local transit options in the jurisdiction of interest when selecting retail centers. A radius of 3 km was drawn around each retail center using 2GIS [33], to identify the catchment area, referred to as the study “sampling area”. The study included sampling areas containing educational facilities (where tobacco sale are prohibited). Effort was made to ensure that sampling areas did not overlap with each other or include areas across PVZs. The shopping mall, intersection, and transit station in each PVZ were identified using an Internet business database (2GIS), and mapping software (Google Maps and Yandex Maps) [34,35]. Using local knowledge, retail centers were selected in areas of the city that had a concentration of tobacco retailers to support expedient data collection by data collectors walking or taking mass transit, or a taxi between venues.

#### Tobacco Retailer-Types Identified

There were three different kinds of retailers that were included in the sample: (1) chain supermarkets (roughly equal number of up market, and mid-low market stores), (2) independently owned markets/convenience stores (including gas stations), and (3) kiosks. Kiosks were explicitly included in the sample because the legislation required these retailers to stop selling tobacco after June 1, 2014.

Ideally, retail venues included in a policy evaluation would be randomly selected from a list of all licensed tobacco retailers. Such lists were not available to the study team, so a walking protocol was developed. The study team set the goal of collecting data from 54 unique retail venues (18 chain super markets, 18 independent markets/convenience stores/gas stations, 18 kiosks) per PVZ (high, average, low), in each of the five cities. This sampling design resulted in 162 tobacco retailers and 270 different retailers per POS type, for a total of 810 tobacco retail locations across the country (Table 3).

**Table 3:** Tobacco retailer sampling goals by city, PVZ, and retailer type.

		Retailer (POS) type			
City	PVZ	Chain supermarket	Independent market/convenience store	Kiosk	Total per city
Moscow	Low	18	18	18	162
	Average	18	18	18	
	High	18	18	18	
St. Petersburg	Low	18	18	18	162
	Average	18	18	18	
	High	18	18	18	
Novosibirsk	Low	18	18	18	162
	Average	18	18	18	
	High	18	18	18	
Yekaterinburg	Low	18	18	18	162
	Average	18	18	18	
	High	18	18	18	
Kazan	Low	18	18	18	162
	Average	18	18	18	
	High	18	18	18	
Total per venue type		270	270	270	Total: 810

#### Point-of-Sale Selection

##### Identification of Chain Supermarkets

Using 2GIS, Google Maps, and Yandex Maps, it was possible to identify and map a comprehensive list of supermarkets and their locations within each of the 9 sampling areas in each city. A list of all chain supermarkets and their classification (up-market, and mid-low market) was created for each sampling area using 2GIS. In all cases, this list was more than the desired 6 supermarkets per sampling area. A random number generator was used to select a total of 6 supermarkets from the lists in each sampling area. When possible, the 6 chain supermarkets included 3 up-market and 3 mid-low market venues. If fewer than 3 high-end supermarkets were located in a sampling area, additional mid- to low-end chain supermarkets were randomly selected to achieve a total of 6 chain supermarket locations per sampling area. Within each sampling area, 3 additional chain supermarkets were randomly selected as back-up retail venues in case any of the primary locations were closed or could not be observed. Data collectors were provided with the lists of randomly selected chain supermarket locations for the 9 sampling areas (3 in each PVZ) in the 5 cities.

##### Identification of Independent Markets/Convenience Stores and Kiosks

After visiting the chain supermarket and collecting relevant observations, data collectors exited the store to identify independent markets (including convenience stores and gas stations), and kiosks (street stalls, often located in underground crossing areas). Researchers identified these venues by following a walking protocol (see Multimedia Appendix 1). First, data collectors exited the supermarket via the main exit/entry point, and would look for an independent market, or kiosk (or underground crossing where kiosks are often located) within eyesight. If there were tobacco retailers located on each side of a street or underground crossing, the POS on the left was selected. If retail venues were only located on the right side of a street or underground crossing, the location on the right was observed. If there was more than one retail venue located on the side of the street or underground crossing being observed (kiosks are often placed in clusters or banks), the closest POS on that side was observed. If data collectors could identify and observe one of each POS type immediately after exiting the supermarket, they would do so and then proceed to the next supermarket location on the list.

If data collectors could not identify an independent market or kiosk immediately after exiting the supermarket, they were instructed to turn left (from the supermarket) and walk, looking for either an independent market or kiosk. Data collectors were instructed to walk in the same direction (walking trajectory) for approximately 5 minutes. If data collectors were able to collect data at both an independent market and kiosk at any point during the 5 minute walking protocol, they could proceed to the next supermarket on the list. If data collectors still needed to observe one or more retail locations after 5 minutes of walking, they were to turn left again (onto a street with vehicle traffic, not a side-street or back-alley), and walk for another 5 minutes, to identify a POS to observe on their path. If data collectors still needed to observe one or more retail locations, they were to turn left again (onto a street with vehicle traffic, not a side-street or back-alley), and this time walk for 10 minutes, to identify a POS to observe on their path. If data collectors still needed to observe one or more retail locations after 10 minutes, they were to turn left again (onto a street with vehicle traffic, not a side-street or back-alley), and walk for another 10 minutes, to identify a POS to observe on their path. Data collectors may have ventured outside of the original 3 km neighborhood radius while following this walking protocol, if observing from a supermarket that was located near the perimeter of the sampling area radius.

After 30 minutes of total walking time (5 minutes, 5 minutes, 10 minutes, and 10 minutes), if data collectors still could not identify an independent market and kiosk or kiosk, they could proceed to the next supermarket on their list. If en route they identified a suitable venue, they could stop there to conduct observations/data collection.

Data collectors were instructed not to observe more than one of the same POS type on the same street. If data collectors could not follow the walking protocol due to the layout of the streets, they were to use their best judgment and document any deviation from the protocol in their daily reports.

##### Revisiting Point-of-Sale Locations

Wave 2 data collection required data collectors to revisit the locations from wave 1. Daily routes were identified using the street location of each POS venue. In the event of no (or unclear) street addresses, reports from wave 1 and photos collected during the initial visit were used to verify the POS and ensure the same location was visited.

### Data Collection Protocol

#### Instrument Content

**Figure 3:**
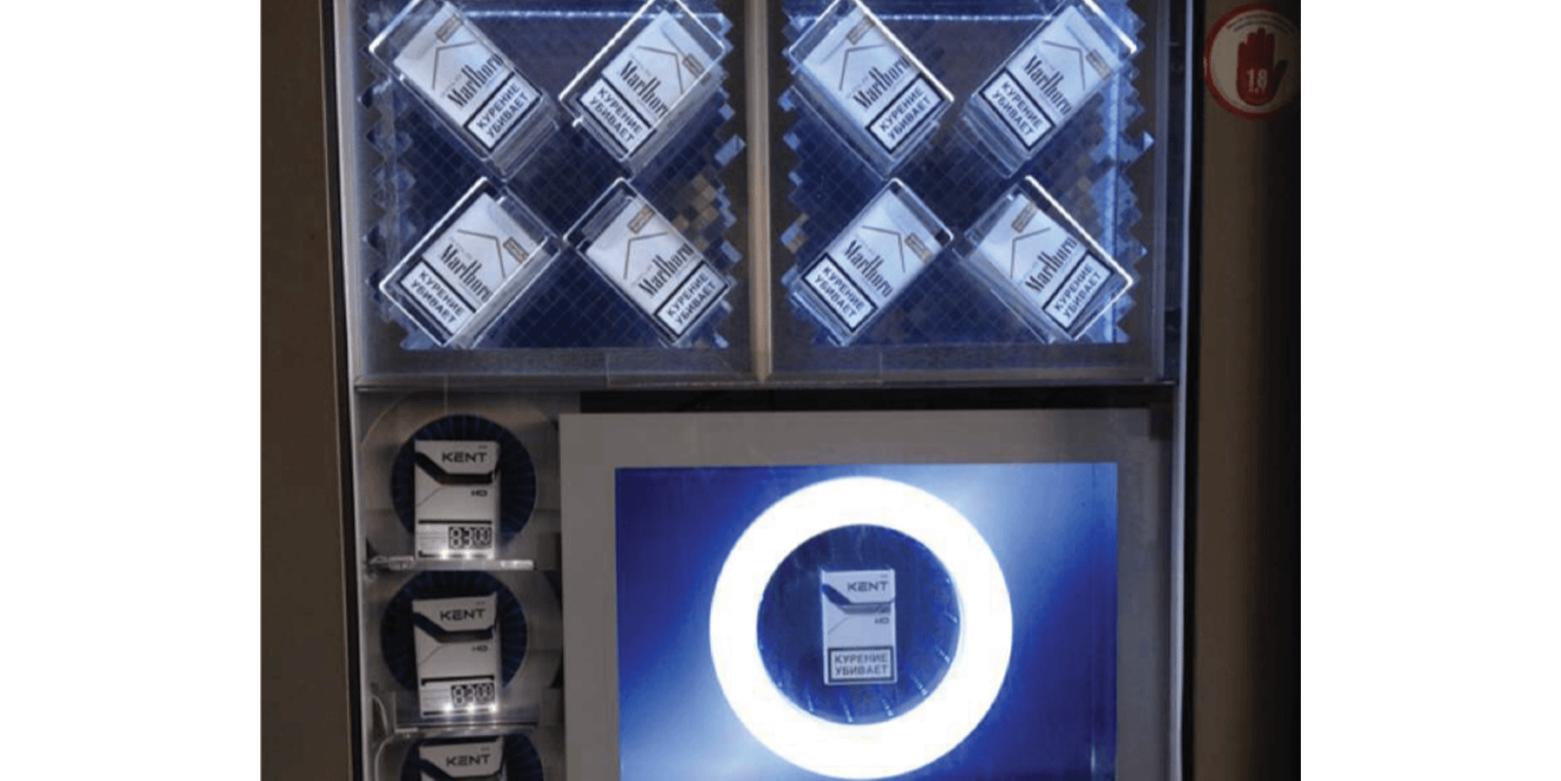
Light-box used for tobacco product "display”.

The survey instrument (observation checklist that contained the items to measure for compliance including product sale, advertisement, promotion, and display) was first developed by thoroughly reviewing the Federal Tobacco Control Law N 15-FZ, focusing on Articles 16 and 19 which regulate TAPS and the retail trade of tobacco products and goods. The instrument was reviewed by CTFK staff and local tobacco control experts including an in-house lawyer in order to ensure that survey questions addressed the specific provisions of the law, as well deficiencies of the law which were known to be exploited with the use of such promotional tactics as light boxes or enlarged packages that advertise a tobacco brand by enhancing the product display (Figure 3 shows this).

Questions that further characterize POS environments, such as availability and display of electronic cigarettes and alcohol, were incorporated in order to measure additional changes in the marketing of these products that may result from the implementation of this policy. Data collected by category and wave are outlined in Table 4.

**Table 4:** Data collected by category and wave.

Category	Wave 1	Added to wave 2
Metadata	•Device identification number •Time data recorded (on device) •Latitude/longitude •GPS accuracy •Record # •Time data uploaded (to cloud database)	
General	•Data collector name •City •PVZ •Sampling area center type (shopping mall, intersection, or transit station) •POS type •Street address	•POS ID# •POS status (open, closed, combined with other POS, or changed POS type) •Does POS still sell tobacco? (yes/no)
TAPS	Presence and offending brand of: •Light boxes •Enlarged packaging •Signage •Imitation tobacco products •Brand stretching •Discounts •Gifts (free or with purchase) •Free distribution of tobacco products •Other advertisement or promotion (open response)	
Product display	Display of tobacco products visible from: •Window on street or underground crossing •Cashier zone •On a power wall •Other display area (open response)	•Compliance with product listing requirements
Image/photograph	•Front/entrance of the POS •Inside POS near display area (optional) •Close-up of product display/advertisement/promotion (optional) •Other POS product display (optional)	
Retail environment	Presence or sale of: •A door for customers to enter/exit •Candies/sweets/snacks in the cashier zone •Alcohol •Gasoline •Electronic cigarettes	

#### Instrument Adaptation for Mobile Data Collection

The survey instrument were inputted into the “Mobile Data Collection” [36] (MDC) software app, using the “Mobile Data Collection Portal” [37]. The MDC software was installed on Blue Dash 4.0 mobile phone devices which were selected for their ability to: function globally (by inserting a subscriber identification module or SIM card from a local network); capture high quality photos; geo-locate; support the MDC app; and for affordability in price. The devices ran on the Android operating system and were configured to display only the MDC app, camera, and toolbar (to control network connection and screen brightness) on the home screen. The MDC software was selected for its ability to capture metadata (specifically the GPS-location feature), collect observations using customized questions and response options including photographs, and upload data in real-time to the cloud-based (Internet-accessible) database, “GIS Cloud” [38]. If the device could not establish network access for real-time upload, data were saved in a queue within the MDC app for automatic uploading once a connection was restored. Fields for capturing photographs of POS entrances (for reference during wave 2 of data collection), product displays, and advertising or promotional activities were added to the survey within the MDC app. Photos of POS entrances were a required field within the survey, while the others were optional. Although mobile phones are a practical tool for discretely collecting data, it may be difficult to capture quality photos of the retail environment in venues that are small and narrow, and in a culture where taking photos in public places may be viewed with suspicion and hostility. While field-testing the protocol during the training period, data collectors were occasionally reprimanded by store clerks or security guards for taking photos. The study team decided to format questions asking for photos of tobacco product displays, advertisements, or promotions as optional fields in order to prioritize the safety of data collectors. The sequence of questions (including photo fields) within the MDC app was designed to match data collectors’ paths as they approached, observed, and departed from the POS in order to minimize observation time and allow for discrete collection of data.

#### Mobile Data Collection

**Figure 4:**
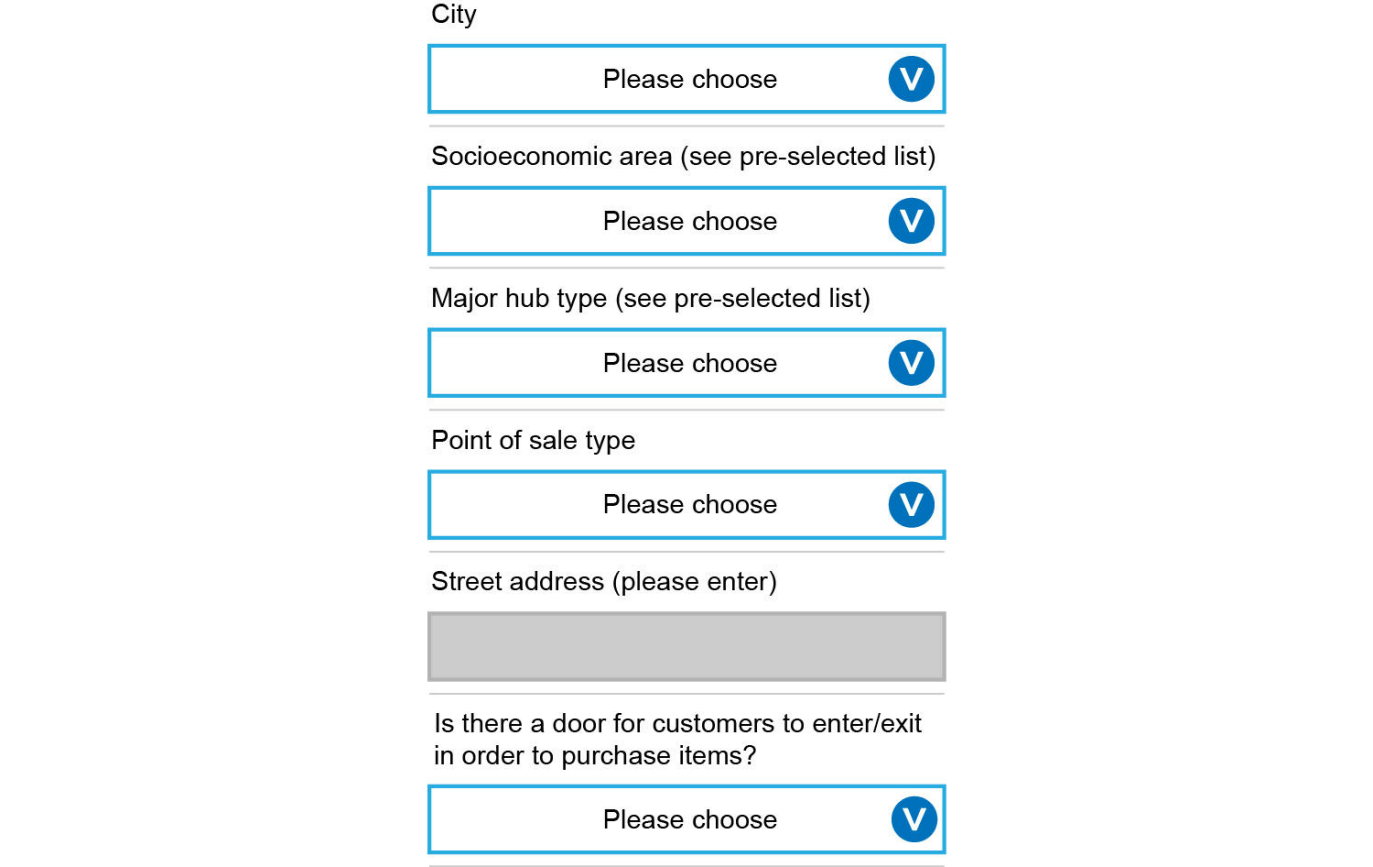
Mobile data collection app interface from mobile phone (general questions).

**Figure 5:**
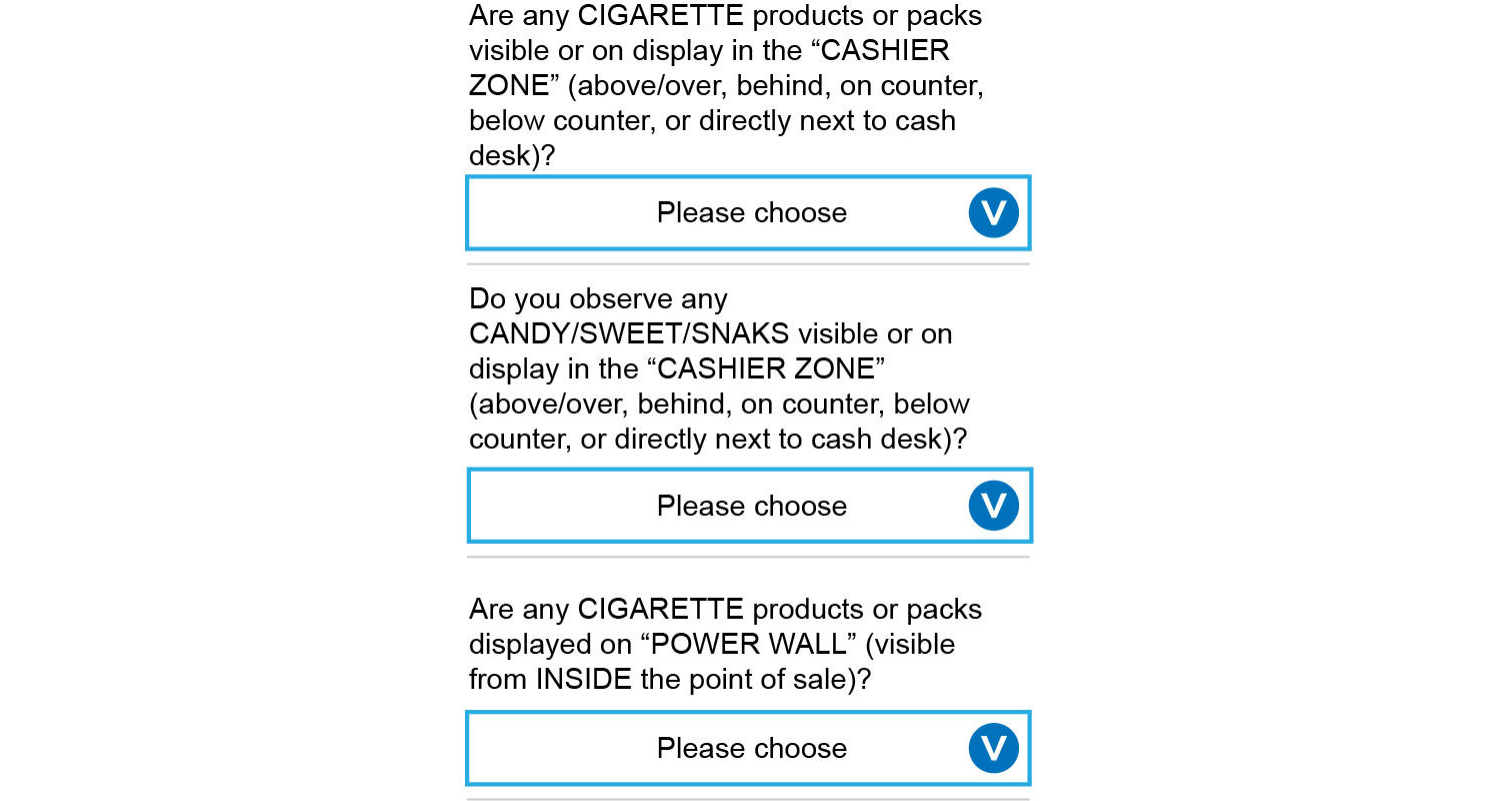
Mobile data collection app interface from mobile phone (product display questions).

Before setting out to observe tobacco retailers, data collectors were to ensure their devices were fully charged, and that SIM cards were loaded and functioning in their mobile phones (to confirm network connectivity). Data collectors were instructed to carry a copy of the protocol, the mobile phone charger, and paper copies of the survey instrument (in case of device or software failure) during each observation day. Before initiating a new record (venue), data collectors were to verify that the GPS and WiFi functions were activated on the mobile phones (in order to optimize geo-location), and to verify their location on the mapping function within the MDC app. Data collectors began by making observations from outside of the POS, before entering the location (if applicable). Data collectors were instructed to behave as customers (using a shopping basket, making small purchases) and to appear as if they were using their mobile phones in a normal manner (texting, looking at a shopping list, playing a game) in order to ensure discretion while extending observation time. If electronic cigarettes were not on display at a particular POS, data collectors were required to ask a salesperson or cashier if they were available for sale as they exited the location. If needed, data collectors were able to use the mobile phone’s default mapping software (Google Maps) to navigate to the supermarkets on their lists, but were encouraged to disconnect the GPS and WiFi functions and reduce screen brightness when possible, in order to conserve battery power. Figures 4 and 5 show the MDC app interface for collecting observational data at a POS.

#### Training and Pilot Testing

Data collectors attended a three-day, in-person training in Moscow given by the research team and were trained in all aspects of the study, including the legal/policy background, data analysis objectives, and intended use of results. Training materials included content on the nature and function of TAPS activities at retail venues and relevant components of the tobacco control law before outlining the sampling framework for each city. Data collectors studied the venue inclusion protocol for identifying and selecting independent markets and kiosks surrounding the selected supermarket locations. A thorough explanation of the survey instrument included definitions and photos of key terms and observation items.

As part of the training, data collectors practiced using the mobile phones provided for data collection in order to familiarize themselves with the device operating system and the electronic version of the survey instrument and response options within the MDC app. The group reviewed strategies for discretely collecting data and taking photos. Training allowed two full days to pilot-test the data collection protocol and instrument, in order to optimize the walking instructions and survey format, and prepare data collectors to record observations.

### Data Management Protocol

In order to verify that data were being correctly recorded (by data collectors) and uploaded (by the MDC app and mobile phone device), data collectors in Russia prepared and submitted daily field reports to the study team in Baltimore, USA. The reports showed the city, PVZ, sampling area type, POS type, POS address, and data collector name for each POS location observed that day, as well as comments addressing any deviations in the walking protocol or observations that may not have been captured within the form. The study team was also able to review data daily by accessing the Geographic Information System (GIS) Cloud database and validate observations by cross-referencing the uploaded data with the daily reports. Cross-referencing the uploaded data with the photos that were captured from retail locations also validated observations.

## Results

### Sampling Approach

#### Wave 1 Sample

Observations were recorded from 780 unique tobacco retail venues in five cities from April 17-May 30, 2014 (Table 5). Researchers found differences in the dispersion/density of retail locations and urban layout/design between cities. During collection, the study team discovered that some sampling areas contained educational or health care facilities, where the sale of tobacco was prohibited (on the premises and within 100 meters). Retail venues located adjacent to these facilities could not be observed if they did not sell tobacco products, which required data collectors to spend additional time and cover a greater distance using the walking protocol. Data collectors also found that kiosks were sometimes located in clusters near sampling area centers rather than dispersed within the 3 km radius of the retail center, and were not identified using the walking protocol. The protocol was adjusted during the collection period to allow data collectors to observe more than one of the same POS type on the same street. There were two additional transit-station sampling areas that were identified for each PVZ in each city, except Novosibirsk, where data collectors were easily able to identify the required POS types and locations. Data collectors visited the additional sampling areas in each city to identify and observe the remaining independent market/convenience stores and kiosks needed that could not be identified by following the walking protocol in the primary sampling areas. Sampling area centers (instead of supermarkets) served as the starting point for data collectors to follow the walking instructions. These minor adjustments were necessary in order to account for the nonuniform distribution of retail venues within the cities. Researchers applying this protocol in other locations should be familiar with the dispersion of retailers in the jurisdiction of interest, and be mindful of adjustments that may confound the stratification of venues, such as between PVZ’s or SES. The use of extra sampling areas to identify retail venues resulted in only 1 location being observed twice, by different data collectors, likely an effect of passing through bordering sampling areas while following the walking protocol. Once identified, the duplicate observation was dropped from the dataset. In the city of Kazan, which is the smallest city by population included in the sample, data collectors were only able to identify 30 of the target 54 kiosks using the walking protocol and extra sampling areas. We expect that the lower number of kiosks observed in Kazan is due to the size of the city and overall availability of retail venues.

#### Wave 2 Sample

The sampling objective for the second wave of data collection was to revisit and observe the same 780 retail locations observed during wave 1. Data collectors were provided with a list of retail venues visited in wave 1, organized by sampling area, that included a POS identification number, retailer type, address, and photo. Data collectors returned to these same locations to collect observations during wave 2 of this study. Of the 780 retail venues observed during wave 1, 779 were revisited during wave 2 of data collection (Table 6); only one POS could not be located (Table 7).

**Table 5:** Retail venues observed in wave 1 of data collection.

Wave 1 locations observed					
		POS type			
City	PVZ	Chain supermarket	Independent market/convenience store	Kiosk	Total per city
Moscow	Low	18	19	19	167
	Average	18	18	18	
	High	23	17	17	
St. Petersburg	Low	17	17	15	156
	Average	18	18	18	
	High	18	19	16	
Novosibirsk	Low	18	18	18	162
	Average	18	18	18	
	High	19	17	18	
Yekaterinburg	Low	18	18	18	162
	Average	19	17	19	
	High	16	19	18	
Kazan	Low	17	18	9	133
	Average	16	17	13	
	High	18	17	8	
Total per venue type		271	267	242	780

**Table 6:** Retail venues observed in wave 2 of data collection.

Wave 2 locations observed					
		POS type			
City	PVZ	Chain supermarket	Independent market/convenience store	Kiosk	Total per city
Moscow	Low	18	18	16	150
	Average	15	15	15	
	High	21	17	15	
St. Petersburg	Low	17	17	11	148
	Average	18	16	18	
	High	17	18	16	
Novosibirsk	Low	18	16	16	148
	Average	18	17	15	
	High	18	16	14	
Yekaterinburg	Low	18	17	18	152
	Average	19	15	15	
	High	16	18	16	
Kazan	Low	17	18	6	122
	Average	15	14	9	
	High	18	17	8	
Total per venue type		263	249	208	720

**Table 7:** Status of retail venues revisited in wave 2 of data collection.

Wave 2 POS status	
Open and still sell tobacco	589
Open and no longer sell tobacco	131
Closed	52
Not observed (failed upload)	7
Not observed (location not found)	1
Total	780

### Data Collection Protocol

#### Wave 1 Data Collection

At the onset of data collection, the research team identified that the use of radio buttons within the survey caused data collectors to accidentally select or change a response option when scrolling down the page to subsequent questions. This issue was resolved by adjusting the response format of the survey questions to provide a drop-down list rather than radio buttons to enter observations. Although four people attended the training, only three of those data collectors were deemed competent enough in the protocol during the pilot test to participate in actual data collection. No further adjustments were required for the data collection protocol during wave 1. Data collectors captured a total of 1815 images during wave 1 of the study, 780 of which were required photos of the venue entrance, and 1035 of which were optional photos of tobacco product displays, advertisements, or promotions.

#### Wave 2 Data Collection

During wave 2 of this study, the survey instrument was duplicated from wave 1 and inputted to the MDC software app following the same mobile adaptation procedure used in wave 1. Only three questions were added to the observation checklist in order to record whether a POS location was still in business, selling tobacco, and whether the newly required product listings were compatible with the law. As a walking protocol was not required during wave 2, data collectors simply navigated to each POS location on their assigned lists and followed the same data collection protocol utilized during wave 1.

Wave 2 data collectors underwent the same training and pilot testing as in wave 1. The data collectors in wave 2 of this study included two staff members who participated in wave 1 and two staff members who were new to the study. Although the walking protocol was not needed for wave 2, the instructions were thoroughly reviewed with the data collection team to support ease of identifying POS locations to revisit. Data collectors also reviewed the newly implemented requirements for product listings that took effect following the June 1^st^ display ban, and this information was provided for reference within the study protocol. During wave 2, the team collected 1277 images from retail venues, 720 of which were required photos of the venue entrance, and 557 of which were optional photos of the newly implemented tobacco product cases, or violations of the law showing display, advertisement, or promotion of tobacco products.

### Data Management Protocol

The research team monitored data collection by reviewing the data as they were uploaded to the cloud database and cross-referencing the uploaded data with the collection schedule and daily reports submitted by each data collector. This detailed oversight allowed the study team to quickly identify and troubleshoot the feature within the layout of the survey that caused data collectors to select incorrect response options. The use of daily reports to validate POS characteristics also helped to identify and correct a glitch in capturing geographic coordinates for each location observed. Due to varying network coverage between the cities, one mobile phone during wave 1 data collection device became “stuck” on a particular set of coordinates in Novosibirsk and continued to record these coordinates for dozens of retail venues in St. Petersburg. The use of reference information allowed us to verify in which city the retailers were actually located and to correct this information within the dataset. A review of POS photos and data also revealed duplicate uploads of five retail locations, which were deleted from the dataset.

During wave 2, the data collection team submitted daily reports in the same format as wave 1, using Google Sheets to update one unique report per city rather than reconciling multiple Microsoft Excel files from each data collector. The daily reports were amended to include fields for recording whether a retail location was still open and selling cigarettes. The study team identified a problem within the MDC software app/GIS Cloud, wherein observations recorded within the mobile survey instrument were not properly uploaded and those fields were left blank. The team made efforts to resolve this issue during the data collection period, but the software development team was unable to address the issue until data collection was nearly completed, resulting in missing data for several observation items during wave 2.

## Discussion

### Principal Findings

The sampling approach, data collection, and data management protocols worked well and only minor revisions were needed during fieldwork to improve the protocols workability. Aspects of this study can be adapted and operationalized for rapid evaluation of POS marketing regulations or other policy assessments in a variety of jurisdictions by NGO’s, local government, or academic researchers. Tailored design of the sampling approach and walking protocol to the local context in Russia was essential to the collection of quality data. A detailed review of the tobacco control law and customization of the survey instrument with the help of local legal experts and advocates resulted in a checklist that captured valuable data for assessing compliance, identifying loopholes in the law, and detecting possible changes in the retail environment that result from policy implementation. Pilot testing of the walking protocol most importantly, as well as the survey instrument, mobile phone technology, and data collection protocol, were essential to optimizing the procedures and survey format for collecting observations quickly and discretely. Future adaptations of these protocols may need to pilot test the walking protocol in each city depending on the scale of the jurisdiction and variation in urban or rural layout and dispersion of local retail venues. Pilot testing in multiple cities would also further inform time required to collect data, and other logistical considerations. Comprehensive training of data collectors, not only in protocols, tools, and technology, but also in the law, purpose of study, remote data management process, intended analysis, and use of study results, prepared data collectors in Russia to execute the protocol, and proactively communicate with the study team in Baltimore to troubleshoot issues in the field.

Vigilance in data management and validation, and close communication between the study coordinators and the field team, were essential to ensuring the success of all protocol components. The use of metadata, photos, and daily reports allowed the study team to identify and solve several problems in real-time during pilot testing and data collection periods, which would have otherwise resulted in the collection of invalid observations and inaccurate geospatial data. If possible, additional pilot testing of this process including a simulation of potential problems that may arise during the influx of data-upload would more thoroughly prepare the study coordinators and field team to address these issues while working remotely and across multiple time zones. Further pilot testing of the data management protocol and MDC software would also inform which metadata and other validation measures to have in place (such as daily reports or daily tests of software and network functioning) to anticipate and circumvent problems that may occur in the field, and allow coordinators to allocate the human resources needed to review uploaded data. The study coordinators should be mindful to anticipate complications and schedule sufficient time to communicate with the field team on a regular basis in order to address and solve issues that may arise with the technology or data collection in general. Coordinators may also want to establish a connection to representatives of the mobile software app selected for data collection, before data collection begins. Even free software programs generally offer basic support for bugs, glitches, and troubleshooting that may be useful when working within a short timeline before a policy is implemented, or another key change takes place in the retail environment. Detailed measures for data management and validation help ensure the integrity of data and study protocols.

### Limitations

The protocols used in this study were limited by lack of availability of comprehensive lists of tobacco retail venues, including the databases we referenced to identify chain supermarkets, which may have been missing some locations. The study also needed to use proxy values for SES; using property values is an adequate proxy for income, but may not overlap with dimensions of education or other predictors of tobacco use. Although the study team may have ventured outside of the defined 3 km sampling radius while collecting observations, the walking protocol kept data collectors within the sampling areas’ corresponding PVZ’s, and did not result in any POS locations being misclassified by PVZ type. Relying on digital technology to collect data can be risky, as the data can be lost or compromised if an error occurs within the mobile phone hardware, app software, or communication network. During wave 1, the study team experienced a problem with the network that recorded inaccurate geographic coordinates for some locations, while 5 duplicate records and 7 failed uploads were identified during the second wave of data collection. When using mobile and cloud-based technology, vigilant monitoring of the dataset and close communication with the field team are imperative to ensuring collection of comprehensive, high quality data. Given the high prevalence of Internet use in Russia, these issues are probably of less concern than they might be on other jurisdictions.

### Conclusions

The use of a walking protocol and mobile technology was helpful for conducting a tobacco control policy evaluation of retail venues and can be adapted for use in other jurisdictions or policy settings. Successful operationalization of a walking protocol requires familiarity with cities and retail environments, and can be useful for evaluations where reliable sampling information, such as lists of retail venues, are not available. The introduction of new technology to these protocols also adds a requirement for thorough training, testing, and very close oversight of data collection. The protocols outlined in this manuscript offer relatively low cost, accessible methods for conducting an expedient evaluation of POS marketing restrictions, retail environments, and policy implementation in general.

## Appendix

Multimedia Appendix 1 Walking protocol animation.

## Abbreviations

apps: applications
CTFK: Campaign for Tobacco Free Kids
GIS: geographic information system
GPS: global positioning system
IGTC: Institute for Global Tobacco Control
MDC: mobile data collection (software application)
NGOs: nongovernmental organizations
POS: point-of-sale (of tobacco products)
PVZ: property-value-zone
Russia: Russian Federation
SES: socioeconomic status
TAPS: tobacco advertising, promotion, and sponsorship
WiFi: wireless local area network
WHO: World Health Organization
