# Supplementary figures and images for: The Development and Piloting of a Mobile Data Collection Protocol to Assess Compliance With a National Tobacco Advertising, Promotion, and Product Display Ban at Retail Venues in the Russian Federation
Source: JMIR Res Protoc. 2016 Aug 31;5(3):e120. doi: 10.2196/resprot.5302 (PMC5023945; doi:10.2196/resprot.5302)

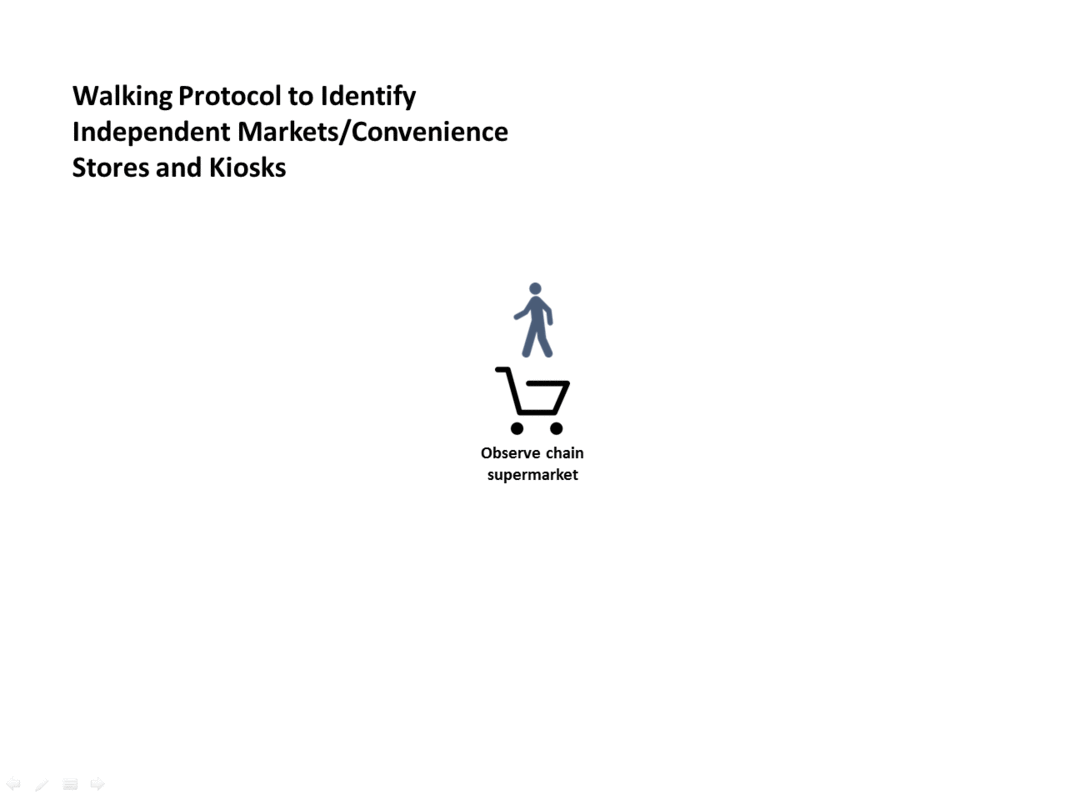

Supplement: Multimedia Appendix 1 [file resprot_v5i3e120_app1.gif]
